# Physical behaviors and their association with type 2 diabetes mellitus risk markers in urban South African middle-aged adults: an isotemporal substitutionapproach

**DOI:** 10.1136/bmjdrc-2022-002815

**Published:** 2022-07-13

**Authors:** Clement N Kufe, Julia H Goedecke, Maphoko Masemola, Tinashe Chikowore, Melikhaya Soboyisi, Antonia Smith, Kate Westgate, Soren Brage, Lisa K Micklesfield

**Affiliations:** 1 Department of Paediatrics, Faculty of Health Sciences University of the Witwatersrand, Johannesburg, Gauteng, South Africa; 2 Epidemiology and Surveillance Section, National Institute for Occupational Health (NIOH), National Health Laboratory Service (NHLS), Johannesburg, Gauteng, South Africa; 3 Non-communicable Disease Unit (NCDU), South African Medical Research Council (SAMRC), Tygerberg, South Africa; 4 MRC Epidemiology Unit, University of Cambridge, Cambridge, UK

**Keywords:** Diabetes Mellitus, Type 2, Insulin Secretion, Insulin Resistance, Obesity

## Abstract

**Introduction:**

To examine the associations between physical behaviors and type 2 diabetes mellitus (T2DM) risk markers in middle-aged South African men and women.

**Research design and methods:**

This cross-sectional study included middle-aged men (n=403; age: median (IQR), 53.0 (47.8–58.8) years) and women (n=324; 53.4 (49.1–58.1) years) from Soweto, South Africa. Total movement volume (average movement in milli-g) and time (minutes/day) spent in different physical behaviors, including awake sitting/lying, standing, light intensity physical activity (LPA) and moderate-to-vigorous intensity physical activity (MVPA), were determined by combining the signals from two triaxial accelerometers worn simultaneously on the hip and thigh. All participants completed an oral glucose tolerance test, from which indicators of diabetes risk were derived. Associations between physical behaviors and T2DM risk were adjusted for sociodemographic factors and body composition.

**Results:**

Total movement volume was inversely associated with measures of fasting and 2-hour glucose and directly associated with insulin sensitivity, basal insulin clearance, and beta-cell function, but these associations were not independent of fat mass, except for basal insulin clearance in women. In men, replacing 30 min of sitting/lying, standing or LPA with the same amount of MVPA time was associated with 1.2–1.4 mmol/L lower fasting glucose and 12.3–13.4 mgl^2^/mUmin higher insulin sensitivity. In women, substituting sitting/lying with the same amount of standing time or LPA was associated with 0.5–0.8 mmol/L lower fasting glucose. Substituting 30 min sitting/lying with the same amount of standing time was also associated with 3.2 mgl^2^/mUmin higher insulin sensitivity, and substituting 30 min of sitting/lying, standing or LPA with the same amount of MVPA time was associated with 0.25–0.29 ng/mIU higher basal insulin clearance in women.

**Conclusion:**

MVPA is important in reducing T2DM risk in men and women, but LPA appears to be important in women only. Longitudinal and intervention studies warranted to provide more specific PA recommendations.

**WHAT IS ALREADY KNOWN ON THIS TOPIC**

- Extensive evidence has shown an inverse association between physical activity and type 2 diabetes risk and report that time spent in sedentary behaviors is a recognized risk factor for type 2 diabetes. Most of these studies have only considered the association between the physical behaviors themselves and type 2 diabetes risk, without also considering the behavior that is replaced for that time. There is also controversy in the literature as to whether the association between physical activity and type 2 diabetes risk is independent of adiposity. It is well recognized that wearable devices provide a more accurate and objective measure of physical behaviors compared with self-report, with limited data combining the signals from two accelerometers to more accurately measure intensity and posture and limited data from Africa.

**WHAT THIS STUDY ADDS**

- Total movement volume was inversely associated with fasting and 2-hour glucose and positively associated with insulin sensitivity, basal insulin clearance, and beta-cell function, but these associations were not independent of fat mass, except for basal insulin clearance in women.
- In men, substituting 30 min of awake sitting/lying, standing, or light intensity physical activity with the same amount of time in moderate-to-vigorous intensity physical activity was associated with lower fasting glucose and higher insulin sensitivity.
- In women, substituting 30 min of awake sitting/lying with the same amount of standing or light intensity physical activity was associated with lower fasting glucose, and substituting 30 min of awake sitting/lying with the same amount of standing was associated with higher insulin sensitivity.
- In women, substituting 30 min of awake sitting/lying, standing or light intensity physical activity with the same amount of time in moderate-to-vigorous intensity physical activity was associated with higher insulin clearance.

**HOW THIS STUDY MIGHT AFFECT RESEARCH, PRACTICE OR POLICY**

- Intervention studies are needed to determine whether sex-specific physical activity recommendations are needed as although moderate-to-vigorous intensity physical activity is associated with lower risk type 2 diabetes risk markers in men and women, light intensity physical activity seems to be beneficial in women only.

## Introduction

The prevalence of type 2 diabetes mellitus (T2DM) is increasing globally, and sub-Saharan Africa (SSA) is projected to have the greatest estimated increase compared with all other regions by 2045.^1^ Within SSA, South Africa (SA) has the highest prevalence of T2DM, with the latest national prevalence for adult men and women at 8% and 13%, respectively.^2^ Extensive evidence reports an inverse association between physical activity (PA) and T2DM risk.^3–5^ Studies have shown that PA of any intensity positively influences glucose regulation and insulin sensitivity in a dose–response manner.^6 7^

Sedentary time is also recognized as a risk factor for T2DM with a systematic review and meta-analysis showing that participants who reported the greatest sedentary time were at a 112% higher relative risk of T2DM compared with those with the lowest sedentary time.^8^ While some studies have shown this association to be independent of PA,^9^ a meta-analysis by Patterson *et al*^10^ showed an increased risk for T2DM with higher levels of total sitting independent of PA.

Time spent in sedentary behavior, PA and sleep are mutually exclusive, and the total minutes available in a day are fixed and finite. Therefore, understanding the beneficial effects of PA depend on the considered aspect of PA and on the activity type displaced.^11^ A recent meta-analysis reported that replacing 30 min of sedentary time with the same amount of time in light intensity physical activity (LPA) was associated with reductions in fasting insulin, waist circumference and all-cause mortality, and replacing sedentary time with moderate-to-vigorous intensity physical activity (MVPA) was associated with reductions in body mass index (BMI), waist circumference, fasting glucose and insulin concentrations, and all-cause mortality.^12^ Furthermore, results from the UK Biobank study (n=4 75 502) reported that replacing sedentary behavior with 30 minutes/day of physical activities or structured exercise was associated with a 6%–31% lower incidence of T2DM 11 years later.^13^ Isotemporal substitution analysis simultaneously models a specific activity and the effects of time in substitution of the activity by another for the same amount of time.^14^ However, the use of isotemporal substitution has been limited to studies including European populations, with a dearth of studies in Africa.

A South African population-based survey reported that only 14.8% were moderately physically active and 27.8% were vigorously physically active and that men were more likely to be physically active whereby women were less likely to engage in moderate as well as vigorous PA.^15^ Few South African studies have explored the association between physical behaviors and T2DM risk.^16–21^ Globally, the majority of evidence reporting the association between PA and health outcomes is based on self-reported PA, which has several limitations.^22^ Wearable devices are increasingly being used and provide a more accurate, objective assessment of sedentary behavior and PA intensity and volume than subjective self-reported measures.^23 24^ Edwardson *et al*^25^ have shown that high accuracy can be obtained using two wearable devices with postural categorization from an accelerometer on the thigh and intensity also using information from an accelerometer on the waist. This has, however, never been undertaken in Africa.

There is controversy as to whether the association between PA and T2DM risk is mediated or independent of adiposity.^4^ This is relevant to the South African context where there is a high prevalence of obesity and where adiposity differs significantly between men and women.^2^

The aim of this study is therefore to examine the association between physical behaviors quantified by combining signals from two accelerometers and risk markers for T2DM in middle-aged men and women from urban South Africa using an isotemporal substitution approach. We hypothesized that replacing 30 min of sedentary time or LPA with the same amount of time in higher intensity behaviors is associated with a reduction in the risk markers for T2DM.

## Materials and methods

### Research design, setting, and participants

**Figure 1:**
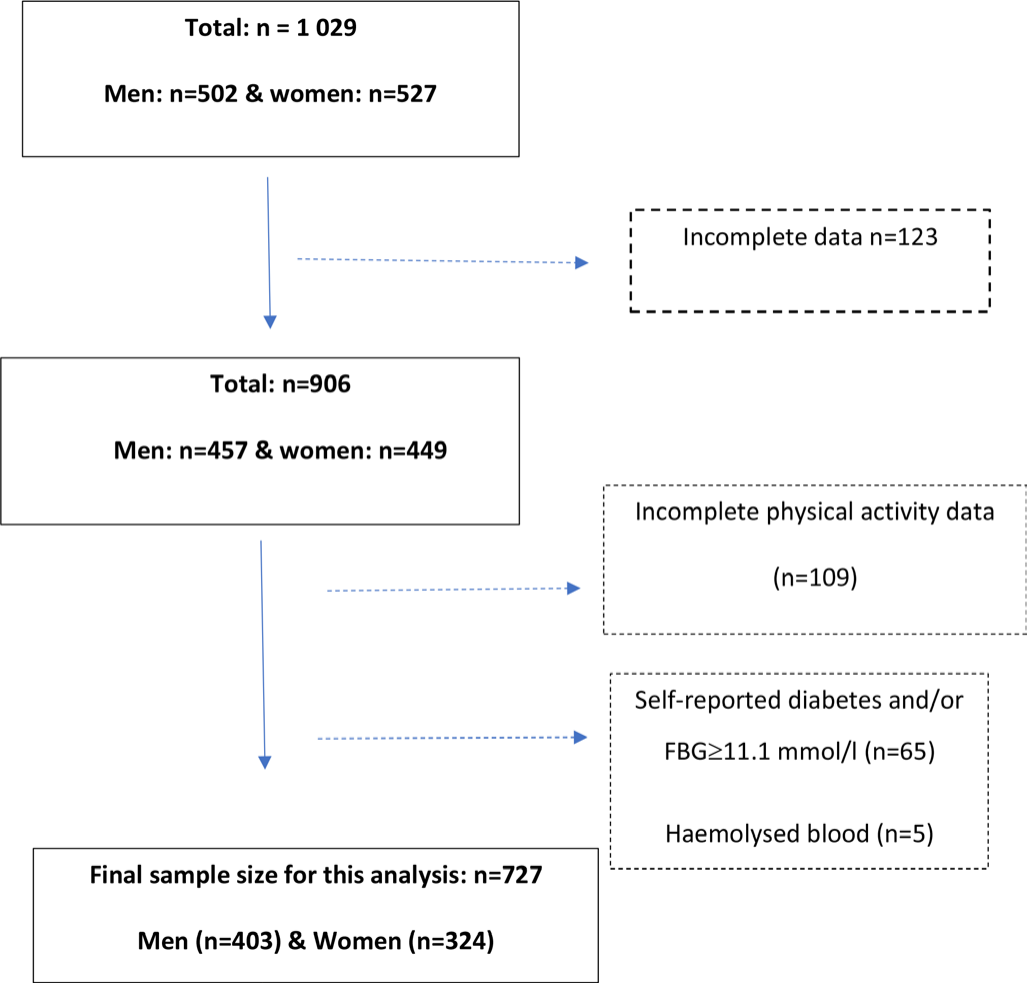
Sample selection flow chart. FBG, fasting blood glucose.

This cross-sectional study used data from the Middle-aged Soweto Cohort collected between January 2017 and August 2018 (502 men and 527 women), as described previously.^26^ For this analysis, complete accelerometry^27^ and oral glucose tolerance test (OGTT) data were available on 727 participants (403 men and 324 women) (figure 1).

## Procedures

### Physical behavior assessment and data processing

Physical behaviors were objectively measured using two accelerometers, an ActiGraph GT3X+ (AG) (ActiGraph, Pensacola, USA) on the right hip, and an activPAL (AP) (PAL Technologies Ltd, Glasgow, UK) on the right thigh. The participants were advised to wear the accelerometers continuously for 7 days and nights including sleep times and the weekend, and only to remove the ActiGraph GT3X+ during bathing or water-based activities. They were also requested to continue with their normal daily activities. Participants received a sleep diary and were asked to record their daily sleep and wake times for the same period.

At the end of the 7 days, participants returned the accelerometers, and raw data were downloaded using the Actilife software (ActiGraph, Pensacola, USA) and actiPAL software (PAL Technologies Ltd). Complete data from ActiGraph GT3X+ and activPAL for 4–7 days was obtained by a combination of processing scripts (PAMPRO) and postprocessing scripts.^28^ The raw triaxial signals from the two accelerometers were calibrated to local gravity^29^ and acceleration and pitch angles converted to minute-by-minute time series. The signals were combined with the self-reported sleep times and reported at participant level to estimate total volume and time spent in postures and intensities of behaviors. The behavior outcomes were summarized as total movement volume (Euclidian norm minus one, ENMO expressed as milli-g (mg)), time spent in sleep, and awake time in sitting/lying, standing, LPA, and MVPA, all in minutes per day (min/day). Details of the objectively measured PA data acquisition, processing, development of the algorithm, and classification are described elsewhere.^26^

### Measures of T2DM risk markers

From each participant, blood samples were drawn after an overnight fast for determination of plasma glucose, serum insulin, C-peptide, and glycated hemoglobin (HbA1c). Participants then underwent a standard OGTT. Participants ingested 75 g anhydrous glucose in 250 mL water, and then 5 mL blood samples were drawn every 30 min for 2 hours. Randox RX Daytona Chemistry Analyser (Randox Laboratories Ltd, London, UK) was used to measure plasma glucose concentrations and D-10 Haemoglobin Analyser (Bio-Rad Laboratories, Inc, USA) was used to measure HbA1c concentrations. Immulite 1000 Immunoassay System (Siemens Healthcare Diagnostics, Tarrytown, New, York, USA) was used to measure serum insulin and C-peptide concentrations.

The homeostasis model assessment of insulin resistance (HOMA-IR) was used to estimate fasting insulin resistance.^30^ Insulin sensitivity was estimated by the Matsuda Index for participants with complete OGTT results and the composite insulin sensitivity index for those with data at 0 and 120 min.^31 32^ Insulin secretion was calculated as the C-peptide index, which was the ratio of the increment in C-peptide to glucose during the first 30 min of the OGTT. The oral disposition index, an estimate of beta-cell function, was calculated as a product of C-peptide index and Matsuda Index.^33^ Basal insulin clearance were estimated as fasting C-peptide/insulin concentrations, and postprandial insulin clearance was estimated from the incremental area under the curve (iAUC) of C-peptide to iAUC insulin using the trapezoidal method.

### Sociodemographic questionnaires

Questionnaires were completed on Research Electronic Data Capture^34^ and included data on age, marital status (married/unmarried), highest level of education attained (no formal schooling/elementary school, secondary school, and tertiary education), 12-item household assets (electricity, television, radio, motor vehicle, fridge, washing machine, telephone/cell phone, microwave, bicycle, tablet/laptop/personal desktop, DSTV/satellite TV, and Mnet) classified into three categories (0–4 for category 1, 5–8 for category 2 and 9–12 for category 3) and employment status (employed/not employed).

### Anthropometry and body composition

Weight was measured with a TANITA digital scale (model: TBF-410, TANITA Corporation, USA) to the nearest 0.1 kg. Height was measured with a wall-mounted stadiometer (Holtain, UK) to the nearest 0.1 cm. BMI was calculated as weight (kg)/(height in m)^2^. Subtotal fat mass (FM; kg) (total FM minus the head) was measured with a Hologic QDR 4500A dual-energy X-ray absorptiometry (DXA) machine (Hologic Inc, Bedford, USA) and analyzed with APEX software V.13.4.2.3 according to standard procedures. Fat mass index (FMI) was calculated as subtotal FM (kg)/height^2^ (m^2^). Abdominal visceral adipose tissue (VAT) and subcutaneous adipose tissue (SAT) areas were estimated from DXA, as described elsewhere.^35^

### HIV/AIDS tests

Pre-HIV and post-HIV counselling was provided and an HIV antibody test, Wondfo One Step HIV–1/2 Whole Blood/Serum/Plasma: Test 2 lines (Guanghu Wondfo Biotech Co, Ltd) was completed for all consented participants, except those previously known as HIV positive. Newly diagnosed HIV positive participants were referred to an HIV clinic for follow-up and retained in the study. Participants were subsequently categorized into HIV negative (HIV−) or HIV positive (HIV+).

### Statistical analysis

Data were analyzed using Stata V.15.1/IC (StataCorp, College Station, Texas, USA). Shapiro-Wilk test and Q–Q probability plots were used to assess distribution and ascertain skewness and kurtosis of the data. Variables were summarized as count (percentages) for categorical data, mean (SD) if normally distributed continuous data, and median (25th–75th percentiles) if not normally distributed continuous data. Student’s t-tests were used to explore the sex differences for normally distributed continuous data, and Mann-Whitney U and Kruskal-Wallis tests were used to compare skewed continuous data. Multivariable robust regression analyses were used to explore the relationship between total movement volume and outcome variables (fasting and 2-hour glucose, insulin sensitivity, basal insulin clearance, and beta-cell function) and covariates that have been shown in the literature to be associated with the outcomes were included. In model 1, age was included as a covariate; in model 2, age, HIV status, education, asset category, and employment were included as covariates; in model 3, FMI was added to covariates in model 2, while in model 4, VAT was included with the covariates from model 3.

Isotemporal substitution modelling was used to estimate the effect of replacing time spent in one physical behavior (sleep, sitting/lying, standing, LPA and MVPA) with another;^14^ specifically, we ascertained the theoretical effect of reallocating 30 min of one physical behavior to 30 min of another physical behavior on the glucose and insulin measures listed previously. Regression coefficients (and 95% CI) represent the replacement of one physical behavior with another, while other behaviors remain constant for the same time. Isotemporal substitution models were adjusted for age, HIV status, education, asset category, and employment (model 2 in total movement volume robust regression analyses) and then subsequently for FMI. Due to marked differences in levels of PA and adiposity between men and women (table 1), all analyses were stratified by sex. A p value of <0.05 was considered significant, and 95% CI was stated.

**Table 1:** Body fat distribution and physical behaviors in men and women

Characteristic	n	Men (n=403)	Women (n=324)	P value
Age (years)	727	53.3±6.2	53.7±5.9	0.450
Height (cm)	727	171.1±6.5	158.3±6.1	<0.001
Weight (kg)	727	73.7±17.7	83.5±18.8	<0.001
BMI (kg/m^2^)	727	25.2±5.8	33.3±7.0	<0.001
Accelerometry (min/day)				
LPA	727	116.5±77.4	121.3±49.6	0.333
MVPA	727	58.6±42.8	34.3±23.2	<0.001
Sitting/lying	675	645.9±124.0	613.9±128.7	0.001
Standing	675	197.5±86.7	258.1±99.3	<0.001
Sleep	727	421.0±84.3	409.5±75.9	0.057
Total movement volume (mg)	727	15.1±6.0	12.4±3.5	<0.001
DXA				
Fat mass (kg)	692	19.2±8.4	36.5±10.7	<0.001
Fat mass index (kg/m^2^)	692	6.6±2.9	14.6±4.2	<0.001
VAT (cm^2^)	692	83.4±42.8	102.7±43.7	<0.001
SAT (cm^2^)	692	194.4±122.0	456.9±149.1	<0.001
Glycemia and insulin dynamics				
Fasting glucose (mmol/L)	725	5.0±1.0	5.1±0.9	0.692
2-hour glucose (mmol/L)	720	5.9±2.5	6.3±2.3	0.064
Fasting insulin (mIU/mL)	723	5.1 (2.1–10.3)	8.9 (5.2–14.5)	<0.001
Fasting C–peptide (ng/mL)	723	1.5 (1.1–2.3)	1.8 (1.3–2.5)	<0.001
HOMA–IR	723	1.1 (0.5–2.3)	1.9 (1.1–3.4)	<0.001
Insulin sensitivity (mgl^2^/mUmin)	719	7.5 (4.0–14.0)	5.0 (3.1–8.2)	<0.001
Insulin secretion (ng/mmol)	628	2.3 (1.3–3.8)	3.1 (1.8–5.1)	<0.001
Basal insulin clearance (ng/mIU)	723	0.29 (0.20–0.40)	0.20 (0.15–0.26)	<0.001
Beta-cell function (mIU/mmol)	610	107.0 (56.6–195.2)	138.2 (61.1–251.3)	0.046

Values for DXA, glycemia and insulin dynamics are mean±SD or median (25th–75th).

BMI, body mass index; DXA, dual-energy X-ray absorptiometry; HOMA-IR, homeostasis model assessment of insulin resistance; LPA, light intensity physical activity; MVPA, moderate-to-vigorous intensity physical activity; SAT, subcutaneous adipose tissue; VAT, visceral adipose tissue.

## Results

### Sample characteristics

Physical behaviors, body fat, and measures of glycemia and insulin dynamics for the whole sample, and men and women separately, are presented in table 1. Men and women were of similar age (~53 years) and a similar proportion were living with HIV (21.1% vs 19.8%, p=0.657). Women had higher BMI and DXA-derived measures of FM, FMI, VAT, and SAT than men (all p<0.001).

There were no sex differences in LPA or sleep time, but men had higher total movement volume (mg) and spent more time in MVPA than women (both p<0.001). Men spent more time sitting/lying and less time standing than women (both p<0.001). There were no sex differences in fasting or 2-hour glucose, while all the measures of insulin dynamics were different between the sexes, with women having higher HOMA-IR, insulin secretion and beta-cell function, and lower insulin sensitivity and basal insulin clearance than men.

### Associations between total movement volume and measures of glycemia and insulin dynamics

In men and women, total movement volume was inversely associated with fasting glucose and positively associated with insulin sensitivity in models adjusted for age, HIV status, and socio-economic status (SES) (table 2). After adjusting for FMI and VAT, these associations were no longer significant. In men only, total movement volume was also inversely associated with 2-hour glucose, but after adjusting for FMI and VAT, this association was no longer significant. In women only, total movement volume was also significantly associated with basal insulin clearance, and this remained significant when adjusting for FMI and VAT. There was no association between total movement volume and beta-cell function in either men or women.

**Table 2:** Multivariable robust regression analyses of total movement volume and measures of glycemic and insulin dynamics

		Men		Women	
**Model 1**	**n**	**β**	**95% CI**	**β**	**95% CI**
Fasting glucose (mmol/L)	725	−**0.014**	−**0.028 to −0.003**	−**0.031**	−**0.054 to −0.009**
2-hour glucose (mmol/L)	720	−**0.033**	−**0.063 to −0.002**	−0.052	−0.109 to 0.004
Insulin sensitivity (mgl^2^/mUmin)	719	**0.240**	**0.138 to 0.343**	**0.162**	**0.049 to 0.275**
Basal insulin clearance (ng/mIU)	723	0.001	−0.001 to 0.003	**0.004**	**0.002 to 0.006**
Beta-cell function (mIU/mmol)	628	0.129	−0.079 to 0.338	0.336	−0.095 to 0.768
**Model 2**					
Fasting glucose (mmol/L)	726	−**0.014**	−**0.027 to −0.0004**	−**0.026**	−**0.050 to −0.002**
2-hour glucose (mmol/L)	720	−**0.033**	−**0.065 to 0.002**	−0.042	−0.103 to 0.018
Insulin sensitivity (mgl^2^/mUmin)	719	**0.199**	**0.096 to 0.301**	**0.138**	**0.022 to 0.255**
Basal insulin clearance (ng/mIU)	723	0.001	−0.002 to 0.003	**0.005**	**0.002 to 0.007**
Beta-cell function (mIU/mmol)	628	0.168	−0.049 to 0.385	0.393	−0.070 to 0.855
**Model 3**					
Fasting glucose (mmol/L)	690	−0.006	−0.019 to 0.008	−0.019	−0.041 to 0.008
2-hour glucose (mmol/L)	685	−0.007	−0.038 to 0.025	−0.011	−0.073 to 0.050
Insulin sensitivity (mgl^2^/mUmin)	684	0.073	−0.015 to 0.161	0.071	−0.059 to 0.201
Basal insulin clearance (ng/mIU)	688	−0.001	−0.003 to 0.002	**0.003**	**0.0001 to 0.005**
Beta-cell function (mIU/mmol)	599	−0.006	−0.227 to 0.215	0.306	−0.194 to 0.807
**Model 4**					
Fasting glucose (mmol/L)	690	−0.004	−0.018 to 0.009	−0.013	−0.037 to 0.011
2-hour glucose (mmol/L)	685	−0.0003	−0.0311 to 0.0317	−0.003	−0.062 to 0.057
Insulin sensitivity (mgl^2^/mUmin)	684	0.053	−0.034 to 0.141	0.072	−0.052 to 0.195
Basal insulin clearance (ng/mIU)	688	−0.001	−0.003 to 0.002	**0.003**	**0.0001 to 0.005**
Beta-cell function (mIU/mmol)	599	−0.061	−0.286 to 0.164	0.236	−0.241 to 0.713

Beta coefficients represent the difference in the outcomes listed per 1 mg difference in movement volume; bold represents significant associations.

Model 1: included age.

Model 2: included age, HIV status, education, asset category and employment.

Model 3: included age, HIV status, education, asset category and employment and FMI.

Model 4: included age, HIV status, education, asset category and employment, FMI and VAT.

FMI, fat mass index; VAT, visceral adipose tissue.

### Isotemporal substitution of physical behaviors in men

Replacing 30 min of sitting/lying, standing or LPA with 30 min of MVPA was associated with 1.2–1.4 mmol/L lower fasting glucose and 12.3–13.4 mgl^2^/mUmin higher insulin sensitivity in men (tables 3 and 4). Replacing 30 min of sitting/lying with the same amount of time standing or in MVPA was associated with 1.4 mmol/L and 3.3 mmol/L lower 2-hour glucose, respectively. Although replacing 30 min of LPA with the same amount of time in MVPA was associated with 3.5 mmol/L lower 2-hour glucose, it was also associated with 1.6 mmol/L lower 2-hour glucose when replaced by standing. Replacing 30 min of sitting/lying and LPA with the same amount of time in MVPA was associated with 17 mIU/mmol and 19.1 mIU/mmol higher beta-cell function, respectively. Replacing physical behaviors was not associated with basal insulin clearance in men.

**Table 3:** Associations of reallocating 30 min of physical behaviors on glycemia for men and women

		β	95% CI	β	95% CI
	**Fasting glucose (mmol/L)**		**Men (n=372)**		**Women (n=301)**
From sitting/lying to:	Sleep	−0.106	−0.561 to 0.348	−0.435	−0.956 to 0.085
	Standing	−0.317	−0.270 to 0.534	−**0.547**	−**0.908 to −0.187**
	LPA	−0.062	−0.528 to 0.040	−**0.790**	−**1.553 to −0.028**
	MVPA	−**1.254**	−**2.148 to −0.359**	0.389	−1.287 to 2.064
From standing to:	Sleep	−0.238	−0.810 to 0.333	0.112	−0.458 to 0.681
	Sitting/lying	−0.132	−0.534 to 0.270	**0.547**	**0.187 to 0.908**
	LPA	−0.194	−0.781 to 0.392	−0.242	−1.114 to 0.629
	MVPA	−**1.386**	−**2.338 to −0.433**	0.937	−0.795 to 2.667
From LPA to:	Sleep	−0.044	−0.583 to 0.494	0.355	−0.510 to 1.220
	Sitting/lying	0.062	−0.404 to 0.529	**0.790**	**0.028 to 1.553**
	Standing	0.194	−0.392 to 0.781	0.242	−0.629 to 1.114
	MVPA	−**1.191**	−**2.243 to −0.139**	1.179	−0.868 to 3.227
From MVPA to:	Sleep	1.147	0.177 to 2.117	−0.824	−2.620 to 0.971
	Sitting/lying	**1.254**	**0.359 to 2.148**	−0.389	−2.065 to 1.286
	Standing	**1.385**	**0.433 to 2.338**	−0.936	−2.669 to 0.795
	LPA	**1.191**	**0.139 to 2.243**	−1.179	−3.227 to 0.868
	**2-hour glucose (mmol/L)**		**Men (n=371)**		**Women (n=298)**
From sitting/lying to:	Sleep	−1.297	−2.325 to −0.269	−1.198	−2.253 to 0.126
	Standing	−**1.385**	−**2.293 to −0.476**	−0.877	−1.791 to 0.035
	LPA	0.199	−0.854 to 1.253	−1.203	−3.134 to 0.726
	MVPA	−**3.261**	−**5.289 to −1.232**	1.102	−3.147 to 5.349
From standing to:	Sleep	0.087	−1.204 to 1.380	−0.320	−1.768 to 1.127
	Sitting/lying	**1.385**	**0.476 to 2.293**	0.877	−0.035 to 1.791
	LPA	**1.584**	**0.259 to 2.909**	−0.325	−2.534 to 1.882
	MVPA	−1.875	−4.033 to 0.282	1.980	−2.415 to 6.376
From LPA to:	Sleep	−1.496	−2.838 to −0.279	0.005	−2.190 to 2.201
	Sitting/lying	−0.199	−1.253 to 0.854	1.203	−0.726 to 3.134
	Standing	−**1.584**	−**2.909 to −0.259**	0.325	−1.882 to 2.534
	MVPA	−**3.460**	−**5.845 to −1.075**	2.306	−2.882 to 7.494
From MVPA to:	Sleep	1.963	−0.237 to 4.165	−2.300	−6.864 to 2.262
	Sitting/lying	**3.261**	**1.232 to 5.289**	−1.102	−5.351 to 3.147
	Standing	1.875	−0.282 to 4.033	−1.980	−6.376 to 2.415
	LPA	**3.460**	**1.075 to 5.845**	−2.306	−7.494 to 2.882

Models are adjusted for age, HIV status, education, asset category, and employment; bold represents significant associations.

LPA, light intensity physical activity; MVPA, moderate-to-vigorous intensity physical activity.

**Table 4:** Associations of reallocating 30 min of physical behaviors on insulin dynamics for men and women

	β	95% CI	β	95% CI
**Insulin sensitivity (mgl** ^**2**^ **/mUmin)**		**Men (n=371)**		**Women (n=297)**
From sitting/lying to: sleeP	2.507	−0.988 to 6.003	−0.986	−3.631 to 1.658
Standing	−0.685	−3.775 to 2.403	**3.229**	**1.404 to 5.054**
LPA	0.366	−3.217 to 3.951	−0.054	−3.907 to 3.798
MVPA	**12.714**	**5.819 to 19.610**	6.866	−1.612 to 15.345
From standing to: sleep	3.193	−1.201 to 7.587	−4.216	−7.105 to −1.326
Sitting/lying	0.685	−2.403 to 3.775	−**3.229**	−**5.054 to −1.404**
LPA	1.052	−3.452 to 5.557	−3.284	−7.692 to 1.124
MVPA	**13.400**	**6.063 to 20.738**	3.636	−5.136 to 12.409
From LPA to: sleep	2.140	−1.997 to 6.279	−0.932	−5.316 to 3.452
Sitting/lying	−0.366	−3.951 to 3.217	0.054	−3.798 to 3.907
Stand	−1.052	−5.557 to 3.452	3.284	−1.124 to 7.692
MVPA	**12.348**	**4.239 to 20.457**	6.920	3.431 to 17.272
From MVPA to: Sleep	−10.207	−17.691 to −2.722	−7.852	−16.960 to 1.254
Sitting/lying	−**12.714**	−**19.610 to −5.818**	−6.866	−15.345 to 1.612
Standing	−**13.400**	−**20.738 to −6.063**	−3.636	−12.409 to 5.136
LPA	−**12.348**	−**20.457 to −4.239**	−6.920	−17.272 to 3.431
**Basal insulin clearance (ng/mIU)**		**Men (n=371)**		**Women (n=300)**
From sitting to: sleep	0.030	−0.052 to 0.113	−0.009	−0.070 to 0.051
Standing	−0.003	−0.077 to 0.069	**0.048**	**0.006 to 0.090**
LPA	0.008	−0.076 to 0.093	0.014	−0.074 to 0.104
MVPA	0.026	−0.137 to 0.190	**0.298**	**0.101 to 0.495**
From standing to: sleep	0.034	−0.069 to 0.138	−0.058	−0.125 to 0.008
Sitting/lying	0.003	−0.069 to 0.077	−**0.048**	−**0.090 to −0.006**
LPA	0.012	−0.094 to 0.119	−0.033	−0.136 to 0.068
MVPA	0.030	−0.143 to 0.204	**0.250**	**0.046 to 0.453**
From LPA to: sleep	0.022	−0.076 to 0.120	−0.024	−0.126 to 0.077
Sitting/lying	−0.008	−0.093 to 0.076	−0.014	−0.104 to 0.074
Standing	−0.123	−0.119 to 0.094	0.033	−0.068 to 0.136
MVPA	0.018	−0.174 to 0.210	**0.284**	**0.043 to 0.524**
From MVPA to: sleep	0.004	−0.173 to 0.181	−0.308	−0.519 to −0.097
Sitting/lying	−0.026	−0.190 to 0.137	−**0.298**	−**0.495 to −0.101**
Standing	−0.030	−0.204 to 0.143	−**0.250**	−**0.453 to −0.046**
LPA	−0.018	−0.210 to 0.174	−**0.284**	−**0.524 to −0.043**
**Beta-cell function (mIU/mmol)**		**Men (n=329)**		**Women (n=256)**
From sitting/lying to: sleep	3.505	−3.687 to 10.698	−1.843	−11.917 to 8.231
Standing	3.827	−2.535 to 10.191	**11.304**	**4.210 to 18.397**
LPA	−2.088	−9.269 to 5.091	2.960	−12.031 to 17.951
MVPA	**16.996**	**2.886 to 31.104**	8.607	−23.341 to 40.556
From standing to: sleep	0.322	−9.519 to 8.874	−13.147	−24.246 to −2.048
Sitting/lying	−3.827	−10.191 to 2.535	−**11.304**	−**18.397 to −4.210**
LPA	−5.915	−15.069 to 3.237	−8.343	−25.643 to 8.955
MVPA	13.168	−1.790 to 28.127	−2.696	−35.778 to 30.384
From LPA to: sleep	5.593	−2.715 to 13.903	−4.803	−21.936 to 12.329
Sitting/lying	2.088	−5.093 to 9.269	−2.960	−17.951 to 12.031
Standing	5.915	−3.237 to 15.069	8.343	−8.955 to 25.643
MVPA	**19.084**	**2.562 to 35.606**	−5.647	−33.324 to 44.618
From MVPA to: sleep	−13.491	−28.962 to 1.980	−10.450	−44.714 to 23.813
Sitting/lying	−**16.996**	−**31.106 to −2.886**	−8.607	−40.556 to 23.341
Standing	−13.168	−28.127 to 1.790	2.696	−30.384 to 35.778
LPA	−**19.084**	−**35.606 to −2.562**	−5.647	−44.618 to 33.324

Models adjusted for age, HIV status, education, asset category, and employment; bold represents significant associations.

LPA, light intensity physical activity; MVPA, moderate-to-vigorous intensity physical activity.

After further adjusting for FMI, the associations between physical behaviors and fasting glucose, insulin sensitivity, and beta-cell function were no longer significant (data not shown). However, replacing 30 min of sitting/lying with 30 min of standing time (1.3 mmol/L, 95% CI 0.4 to 2.2, p=0.003) and replacing 30 min of standing with the same amount of LPA time (1.7 mmol/L, 95% CI 0.4 to 3.0, p=0.007) both remained significantly associated with 2-hour glucose after adjusting for FMI.

### Isotemporal substitution of physical behaviors in women

Replacing 30 min of sitting/lying with standing or LPA were associated with 0.5 mmol/L and 0.8 mmol/L lower fasting glucose, respectively, but were not associated with 2-hour glucose. Replacing 30 min of sitting/lying with the same amount of time standing was associated with 3.2 mgl^2^/mUmin higher insulin sensitivity, 0.05 ng/mIU higher basal insulin clearance, and 11.3 mIU/mmol higher beta-cell function. Replacing 30 min of sitting/lying or standing or LPA with the same amount of time in MVPA was associated with 0.25–0.30 ng/mIU higher basal insulin clearance.

After further adjusting for FMI, the associations with fasting glucose, 2-hour glucose, and insulin sensitivity were no longer significant (data not shown). In contrast, the significant associations with basal insulin clearance were maintained when 30 min of sitting/lying (0.21 ng/mIU, 95% CI 0.41 to 0.02, p=0.029) or standing (0.19 ng/mIU, 95% CI 0.39 to 0.002, p=0.048) were replaced by MVPA. Replacing 30 min of sitting/lying with the same amount of standing was also still associated with higher beta-cell function (8.68 mIU/mmol, 95% CI 0.93 to 16.44, p=0.028) after adjusting for FMI.

## Discussion

This study in a middle-aged African population of men and women showed that PA was associated with lower risk for T2DM. In support of our hypothesis, we showed that replacing 30 min of sedentary time or LPA with the same amount of time in higher intensity behaviors was associated with a reduction in the risk markers for T2DM. Specifically, substituting 30 min of awake sitting/lying, standing or LPA with the same amount of time in MVPA in men was associated with lower fasting glucose and higher insulin sensitivity, both well-accepted indicators of T2DM risk. In women, just replacing 30 min of sitting/lying with the same amount of time standing was associated with lower fasting glucose and higher insulin sensitivity. We have also shown that the associations between physical behaviors and these measures of T2DM risk are mediated by adiposity in both men and women.

In both men and women, our study showed that total movement volume was associated with lower fasting glucose and higher insulin sensitivity, and in men only was also associated with lower 2-hour glucose, although none of these associations remained significant after adjusting for adiposity. PA through its effects on multiple organs and systems is associated with improved insulin sensitivity and glycemia.^5 36^ Data from the EPIC-InterAct case–control study of incident T2DM reported a significant reduction in the risk of developing T2DM in men and women who had higher levels of PA.^37^ Indeed, epidemiological studies have shown that PA reduces the risk of insulin resistance and T2DM in healthy individuals and appropriate exercise training is an effective intervention for individuals at risk of T2DM.^38 39^ PA can reduce the risk of T2DM in men and women with high BMI and elevated glucose levels. Even relatively modest exercise can stimulate immediate and persistent insulin sensitivity the next day in adults at risk of T2DM.^40^ The men in our study completed on average 410 min of MVPA/week while average MVPA/week in the women was 240 min, which may account for the additional effect of PA on postprandial glucose uptake, with skeletal muscle being the major site of uptake. Extensive literature has explored the association between objectively measured PA and measures of T2DM risk.^4 8 9^ Not all of these studies have accounted for adiposity, while others have found the association to be mediated by adiposity,^36^ and still other studies have found the significant association between PA and T2DM remains after adjusting for adiposity.^37 41^ The differences in the results may be due to marked heterogeneity in research designs and assessment of physical behaviors and T2DM risk. In our study, the associations between PA and T2DM risk in both men and women were mediated by total adiposity. Balkau *et al*^42^ reported that objectively measured daily total activity and the association with insulin sensitivity in healthy adult men and women between 30 and 60 years remains even after adjusting for overall BMI or abdominal adiposity.

Using the combination of signals from two accelerometers for objective measurement of physical behaviors, we used isotemporal substitution to account for the displacement of time in particular behaviors within a 24-hour day.^11 14^ We showed that replacing sitting/lying with standing time was only associated with lower 2-hour glucose in the men, while in women, replacing sitting with the same amount of time standing was associated with improvements in fasting glucose, insulin sensitivity, insulin clearance and beta-cell function. This difference when modeling the hypothetical effect of posture change resulting in all aspects of insulin dynamics being affected in women compared with only postprandial glucose in men may be due to the higher adiposity in women. An Australian study in men and women (36–80 years) using the isotemporal substitution approach showed that replacing sitting with standing for 2 hours/day was associated with 2% lowering of fasting plasma glucose in both men and women.^43^ These differences may be accounted for by the vast disparities in adiposity between men and women in our study compared with the study in Australia.

Replacing sedentary time, that is, sitting or standing with movement, irrespective of intensity, has been associated with a wide array of health benefits. Yates *et al*^44^ reported that reallocating 30 min per day of sedentary time to LPA and MVPA was associated with a 5% and 18% difference in insulin sensitivity (Matsuda-ISI) in adult men and women of average age 65 years after adjusting for ethnicity, sex, age, medication, social deprivation, and BMI. In a South African study, LPA has been shown to be associated with reduced cardiovascular disease risk^45^ and has also been included in the recent WHO recommendations for PA.^46^ Interestingly in this study, the only significant associations when replacing sedentary behavior with LPA was a decrease in fasting glucose in the women only when replacing sitting with LPA, and an increase in 2-hour glucose in the men when replacing standing with LPA. The men and women in this study spent an average of 2 hours a day in LPA, and previous studies in similar populations have shown that this is largely time spent in incidental activity or walking for transport rather than leisure time activity, which is typically low in populations from low-income and middle-income countries.^18 47^ As physical behaviors have been accurately measured in this study by combining the signals from two accelerometers, it can be concluded that time spent in LPA in this population is not sufficient to influence diabetes risk significantly.

Time spent in higher intensity PA, in many studies described as MVPA, has been repeatedly shown in the literature to be associated with lower T2DM risk.^3 6 48^ In this study replacing sitting/lying, standing and LPA with MVPA was associated with improvements in the measures of T2DM risk in men including a decrease in fasting glucose and 2-hour glucose and an increase in insulin sensitivity and beta cell function. In contrast in the women, replacing sitting/lying, standing and LPA with MVPA was only associated with an increase in basal insulin clearance. Although we did not specifically examine time spent in vigorous intensity PA, previous research has shown that SA men spend more time in higher intensity activity than women,^49^ which may have explained the greater associations between MVPA and diabetes risk in men than women. Another explanation may be the ‘legacy effect’ of earlier patterns of activity, which have been shown to be higher in men compared with women throughout adolescence in a longitudinal South African cohort^47^ and which may then result in alterations in muscle physiology and improved cardiorespiratory fitness that may be maintained into adulthood. This is consistent with the higher levels of cardiorespiratory fitness in young adult men compared with women from Soweto,^49^ and another study from Cape Town that reported very low levels of cardiorespiratory fitness in young women.^45^ Indeed, cardiorespiratory fitness, and not objectively measured MVPA, has previously been associated with higher insulin sensitivity in black African women.^45^ Results from an exercise intervention study in black African women with obesity and low baseline levels of cardiorespiratory fitness demonstrated that 12 weeks of intensive training only resulted in a small improvement in insulin sensitivity with no changes in glycemia.^50^ This may be explained by low levels of fitness and high levels of obesity and insulin resistance in these women.^45^

Another novel finding of the study was the improvement in insulin clearance when replacing sitting/lying, standing, and LPA, with MVPA, in women only. Previous research has shown that black South African women have low insulin clearance compared with their European counterparts, which contributes to their characteristic hyperinsulinemia.^51^ Furthermore, we showed that women had lower insulin clearance compared with men. It is still unclear if low insulin clearance and/or the resultant hyperinsulinemia is a compensatory response to low insulin sensitivity or is the driver of T2D in the black African women.^51^ Nonetheless, this is the first study to show that MVPA is associated with higher insulin clearance, independent of the effects of adiposity, and that this association is specific to women and not observed in men. Prospective studies are required to investigate whether an increase in insulin clearance, and consequently a reduction in hyperinsulinemia, does confer reduced risk from T2D in black Africans.

The strengths of this study included the combination of signals from two accelerometers for objective measurement of physical behaviors. Most studies have used regression analysis to determine the associations between PA and diabetes risk. Our study used isotemporal substitution and 30 min as this links closely with public health recommendations. These results highlight the importance of measuring different PA behaviors, which are differentially associated with glycemia and insulin dynamics. Notably, we included both men and women and showed differences in the relationship between PA behaviors and diabetes risk. The cross-sectional design is a limitation of the study. Self-reported sleep diaries are prone to recall bias, and social desirability coupled with reporting of time in bed, rather than sleep duration, may introduce misclassification. However, the accelerometry adjunct to sleep diaries assisted in verifying the sleep classification.

In conclusion, this study provides novel evidence on the potential benefits of engaging in more active behaviors on risk factors for T2DM in men and women. This is critical in public health given the high amount of time spent sitting/lying and standing in our study population. Furthermore, longitudinal and intervention research is required to determine whether the effects of different intensity physical behaviors are sex specific and need to be taken into account when designing public health interventions to reduce non-communicable disease risk.

## Data Availability

Data are available on reasonable request.
